# Brain macrostructure correlates of financial altruism in older adults without dementia

**DOI:** 10.1007/s11682-026-01133-x

**Published:** 2026-03-17

**Authors:** Laura Fenton, Gali H. Weissberger, Lauren E. Salminen, Anya Samek, Daisy T. Noriega-Makarskyy, Cassidy P. Molinare, Jordan Williams, Emma Oyen, Paige Kim, Jenna Axelrod, Laura Mosqueda, Hussein Yassine, S. Duke Han

**Affiliations:** 1 Department of Psychology, Arts, and Sciences, USC Dornsife College of Letters, Los Angeles, CA USA; 2 The Department of Social and Health Sciences, Bar-Ilan University, Raman Gat, Israel; 3 Mark and Mary Stevens Neuroimaging and Informatics Institute, Keck School of Medicine of USC, Los Angeles, CA USA; 4 Rady School of Management, UC San Diego, San Diego, CA USA; 5 Department of Family Medicine, Keck School of Medicine of USC, Alhambra, CA USA; 6 Department of Neurology, Keck School of Medicine of USC, Los Angeles, CA USA; 7 Department of Psychology Seeley G. Mudd Building, University of Southern California, 3620 S. McClintock Avenue, Room 501, Los Angeles, CA 90089-1061 USA

**Keywords:** Aging, Decision making, Older adults

## Abstract

Greater financial altruism (i.e., the tendency to give money to an anonymous individual) has been previously associated with poorer performance on neuropsychological tests sensitive to cognitive changes occurring in early Alzheimer’s disease (AD). While these findings provide initial support for the idea that financial altruism may be associated with early AD-related brain changes, no study has used MRI to investigate structural brain correlates of financial altruism in older adults. To address this gap in the literature, the current study investigated associations between brain macrostructure and financial altruism in 101 older adults free from dementia (mean age = 68.45±7.15, mean education = 16.61±2.14, 76% female, 73% White) using a high-field 7 Tesla MRI. Results revealed no significant associations between financial altruism and a priori regions of interest (entorhinal cortex, hippocampus, ventromedial prefrontal cortex, dorsolateral prefrontal cortex, and temporoparietal junction) in the entire sample. However, there was a significant interaction between financial altruism and income, whereby greater financial altruism was associated with lower hippocampal volume in those with a reported income below $100,000. Collectively these results suggest that financial altruism in the context of lower income, but not financial altruism in general, may reflect suboptimal brain aging, and perhaps greater risk of conversion to AD.

**Supplementary Information:**

The online version contains supplementary material available at 10.1007/s11682-026-01133-x.

## Introduction

A growing body of research suggests overlap between brain regions and networks involved in decision making and those affected by Alzheimer’s disease (AD)-related neuropathology. Because of this, financial exploitation vulnerability due to impaired decision making may be an early behavioral manifestation of preclinical AD (Boyle et al., 2019; Fenton et al., 2022; Kapasi et al., 2021; Lamar et al., 2024). One aspect of decision making that may increase risk for exploitation is an increased tendency for financial altruism (i.e., the tendency to give money to an anonymous individual), which can be measured using a behavioral economics measure known as “The Dictator Game”. In this game, the “dictator” is given a sum of money and has the option to share any amount with an anonymous individual (the “recipient”) (Kahneman et al., 1986). The recipient cannot refuse the dictator’s offer, and according to economic theories related to self-interest and rationality, the dictator should always keep the entire dollar amount for themselves (Fehr & Fischbacher, 2002; Guala & Mittone, 2010). Giving behavior in this paradigm has been described as financial altruism, as it confers economic benefit to the recipient at a cost to the dictator (Fehr & Fischbacher, 2003).

Previous studies show that older adults typically behave more financially altruistically than younger adults (Engel, 2011; Kettner & Waichman, 2016; Rosi et al., 2019), but the mechanisms are unclear. A meta-analysis of aging and altruism found that demographic factors (e.g., income, education, sex) did not explain age-related differences in altruism (Sparrow et al., 2021). While age-related shifts in prosociality have often been conceptualized in terms of motivation (Mayr & Freund, 2020), prior work from our lab supports a potential role of cognitive resources. Specifically, our work found that cognitively unimpaired older adults who exhibited higher financial altruism performed worse on cognitive tests sensitive to brain changes that occur in early AD (Weissberger et al., 2022).

In younger adults, structural integrity of the temporoparietal junction and dorsolateral prefrontal cortex (dlPFC) has been associated with financial altruism. In one study where participants allocated money between themselves and an anonymous individual, gray matter volume in the right temporoparietal junction was positively associated with altruistic behavior (Morishima et al., 2012). The authors suggest the temporoparietal junction’s role in perspective taking (Saxe et al., 2004) may increase altruism through the ability to consider the mental state of the recipient. In a second study, altruistic behavior was negatively associated with cortical thickness of the dlPFC (Yamagishi et al., 2016) – an important structure for strategic reasoning and impulse control (Steinbeis et al., 2012). These findings align with evidence that individuals are intuitively prosocial and must inhibit innate prosocial tendencies to act strategically (i.e., maximize personal gains) (Zaki & Mitchell, 2013). However, no study has assessed whether these associations also occur in older adults.

The current study addressed this gap by investigating associations between financial altruism and brain macrostructure in older adults free from dementia. The goal of the study was to identify neuroanatomical correlates of financial altruism in older adults and to determine whether regions vulnerable to neurodegenerative disease (e.g., AD) may be implicated, as suggested by our prior work. Clarifying these relationships may help determine whether the increased financial altruism often observed in older adults reflects normative motivational shifts or early brain changes associated with pathological aging. To investigate the specificity of this measure to detect AD-related brain changes, we focused on five a priori regions of interest (ROIs): the entorhinal cortex (ERC) and hippocampus, ventromedial prefrontal cortex (vmPFC), dlPFC, and temporoparietal junction. The ERC and hippocampus are vulnerable to atrophy very early in AD (Berron et al., 2021; Dickerson et al., 2001; Pini et al., 2016; Planche et al., 2022). Although neither region has been studied in direct connection with altruism, previous work from our group identified a negative association between ERC thickness and financial exploitation vulnerability (Fenton et al., 2024), and a negative association between financial altruism and verbal memory – a function subserved by the hippocampus (Weissberger et al., 2022). The vmPFC is highly involved in making decisions that require valuation of options (Grabenhorst & Rolls, 2011) and the dlPFC and temporoparietal junction were previously associated with altruism when measured via Dictator Game paradigms in younger adults. We hypothesized that greater financial altruism would be associated with lower thickness in the ERC, vmPFC, and dlPFC, lower volume of the hippocampus, and greater thickness in the temporoparietal junction.

## Methods

### Participants

The study included 101 participants (mean age = 68.48±7.13, mean education = 16.60±2.4, 77% female, 73% White) recruited from the greater Los Angeles area through community outreach events, local senior centers, and the Alzheimer Prevention Registry (Langbaum et al., 2020). Participants were required to be aged 50 or older, fluent in English, and free from significant cognitive impairment (i.e., score of 21 or above on a modified version of the 5-minute Montreal Cognitive Assessment (Wong et al., 2015)). Exclusion criteria included significant neurological or psychiatric illness, and current or previous substance use problems. The University of Southern California Institutional Review Board (HS-20-01024) approved all study procedures.

### Measures

#### Behavioral economic measures

To assess financial altruism, participants were handed a piece of paper and instructed, “In this part, you are matched with another person, Person B. Person B is participating in this part online. This part is totally anonymous: you will not know who Person B is, and Person B will never know who you are. You have the opportunity to send a portion of your $10 to Person B. You can send any amount between $0 and $10. Any amount you keep will be added to your earnings at the end of the study today. Any amount you send will be added to Person B’s earnings at the end of the study today.” Participants placed a check mark next to the amount of their $10 they would like to send to Person B. To minimize experimenter demands, the research associate administering the task explicitly looked away while they selected their choice. One participant was excluded due to missing data.

#### Neuroimaging measures

Structural brain MRI data were collected using a 7 Tesla scanner (Siemens Magnetom) with 8-channel head coil through the USC Center for Imaging Acquisition (CIA). Scans were T1 3D MPRAGE images with the following parameters: TE = 2.95ms, TR=2200ms, 240 sagittal slices, acquired voxel size (avs) = 0.7 mm x 0.7 mm x 0.7 mm. Due to the stronger intensity inhomogeneity of the 7T data, images were first bias-corrected using SPM12’s unified segmentation algorithm (Ashburner & Friston, 2005). FreeSurfer version 7.1.0’s high resolution subcortical and cortical pipelines were used (Zaretskaya et al., 2018) to process each image. Regional cortical thickness and volume measures were generated using the Desikan Killiany atlas (Desikan et al., 2006). Each participant’s output was visually inspected and any errors were manually corrected. Nine participants were excluded due to segmentation errors remaining following corrections, resulting in the final sample size of 101. Regions were averaged across hemispheres. ROIs included thickness in the ERC, dlPFC, vmPFC, and temporoparietal junction, and volume of the hippocampus. Thickness measures were selected for cortical ROIs based on prior evidence that thickness is more sensitive to AD-related brain changes than volume, particularly in temporal and parietal regions.

#### Income measure

Income was assessed via responses to the question: “Which category represents the total combined income of all members of your family (living in your house) during the past 12 months? This includes money from jobs, net income from business, farm or rent, pensions, dividends, interest, Social Security payments and any other monetary income received by members of your family who are 15 years of age or older.” There were 16 response categories, ranging from “Less than $5000” to “$150,000 or more.”

### Statistical analyses

Statistical analyses were conducted using R version 4.4.3. Inspection of the altruism variable revealed a multimodal distribution, with peaks at 0, 5, and 10, and inspection of diagnostic plots revealed violations of linear model assumptions (e.g., non-normality of residuals and heteroscedasticity). A Box-Cox analysis suggested a log transformation. However, diagnostic plots continued to show violations of key assumptions after the transformation. Therefore, altruism was treated as a categorical variable, with participants separated into one of three groups: (1) those who gave more money to Person B (i.e., more than $5) than they kept for themselves were included in the “Gave More” (*GM*) group (*N* = 25), (2) those who gave less money to Person B (i.e., less than $5) than they kept for themselves were included in the “Gave Less” (*GL*) group (*N* = 48), and (3) those who split the money equally (i.e., gave $5 to Person B) were included in the “Gave Equally” (*GE*) group (*N* = 28).

General linear models (GLMs) adjusted for age, sex, education, and income were used to examine differences in ROI metrics between groups. Analyses of hippocampal volume were further adjusted for total intracranial volume. Type III sums of squares were used to evaluate effects of altruism group. Pairwise comparisons using estimated marginal means with Tukey’s HSD correction were performed for any significant group effects.

Due to the wide age range of the sample (53–83), additional GLMs including an interaction term for age and altruism group were conducted. To explore any significant interaction effects of age and altruism group, we divided the sample by age using a median split (age 70+ and age <70), and used GLMs stratified by age to examine differences in ROIs between altruism groups. Post-hoc analyses were conducted to explore the interactive effect of income and altruism group on ROIs. To explore any significant interaction effects of income and altruism group, we divided the sample by income category using a median split ($100,000+ and less than $100,000), and used GLMs stratified by income to examine differences in ROIs between altruism groups.

As post-hoc exploratory analyses, separate GLMs including each Desikan-Killiany Atlas cortical thickness ROI and each subcortical ROI (averaged across hemispheres) as the outcome variable were conducted. Findings were corrected for multiple comparisons using the false discovery rate (FDR) method. All models were adjusted for age, sex, education and income, and models with subcortical ROIs were further adjusted for intracranial volume.

## Results

The final sample consisted of 101 older adults (See Table 1 for participant characteristics). Altruism group (i.e., *GM*, *GL*, *GE*) characteristics are also summarized in Table 1. There was a significant difference in income between the *GM* and *GL* groups, such that those in the *GM* group had significantly higher income (*p* = 0.01).

**Table 1:** Sample characteristics

Variable	Entire Sample (*N* = 101)	Gave More (*N* = 25)	Gave Less (*N* = 48)	Gave Equally (*N* = 28)	More vs. Less vs. Equally (*p*-value)
Age, M (SD)	68.45 (7.15)	69.56 (8.5)	67.67 (6.75)	68.79 (6.62)	*p* = 0.54
Sex (% female)	76%	88%	77%	68%	*p* = 0.23
Education, M (SD)	16.61 (2.14)	17.16 (1.75)	16.35 (2.3)	16.57 (2.17)	*p* = 0.31
Race					*p* = 0.96
% White	73%	72%	73%	75%	
% Asian	16%	12%	17%	18%	
% Black	6%	8%	6%	4%	
% Other	2%	4%	2%	0%	
% Unknown	3%	4%	2%	3%	
Ethnicity					*p* = 0.44
% Hispanic	95%	92%	98%	93%	
% Non-Hispanic	5%	8%	2%	7%	
Income					***p*** **= 0.01**
% $150,000 or more	24%	52%	13%	18%	
% $100,000 – $149,999	22%	12%	31%	14%	
% $50,000 – $99,999	33%	28%	29%	46%	
% Less than $50,000	18%	8%	23%	18%	
% Unknown	3%	0%	4%	4%	
MMSE, M (SD)	28.48 (1.49)	28.36 (1.52)	28.38 (1.55)	28.75 (1.38)	*p* = 0.62

Differences between groups were assessed using One-Way ANOVA for continuous variables and Chi-square or Fisher's Exact Test for categorical variables

*MMSE* Mini-Mental State Exam

No significant differences in ROIs were observed between altruism groups. Models including an interaction term for age and altruism group revealed a trend on vmPFC thickness (F = 2.86, *p* = 0.06), but in models stratified by age, altruism group was not significantly associated with vmPFC thickness. Exploratory pairwise comparisons revealed that, in the older group, those in the *GM* group had marginally greater thickness of the vmPFC (M difference = 0.092, 95% CI [0.015, 0.169], *p* = 0.054) compared to those in the *GE* group (Supplementary Fig. 1). No other significant differences were observed.

**Fig. 1:**
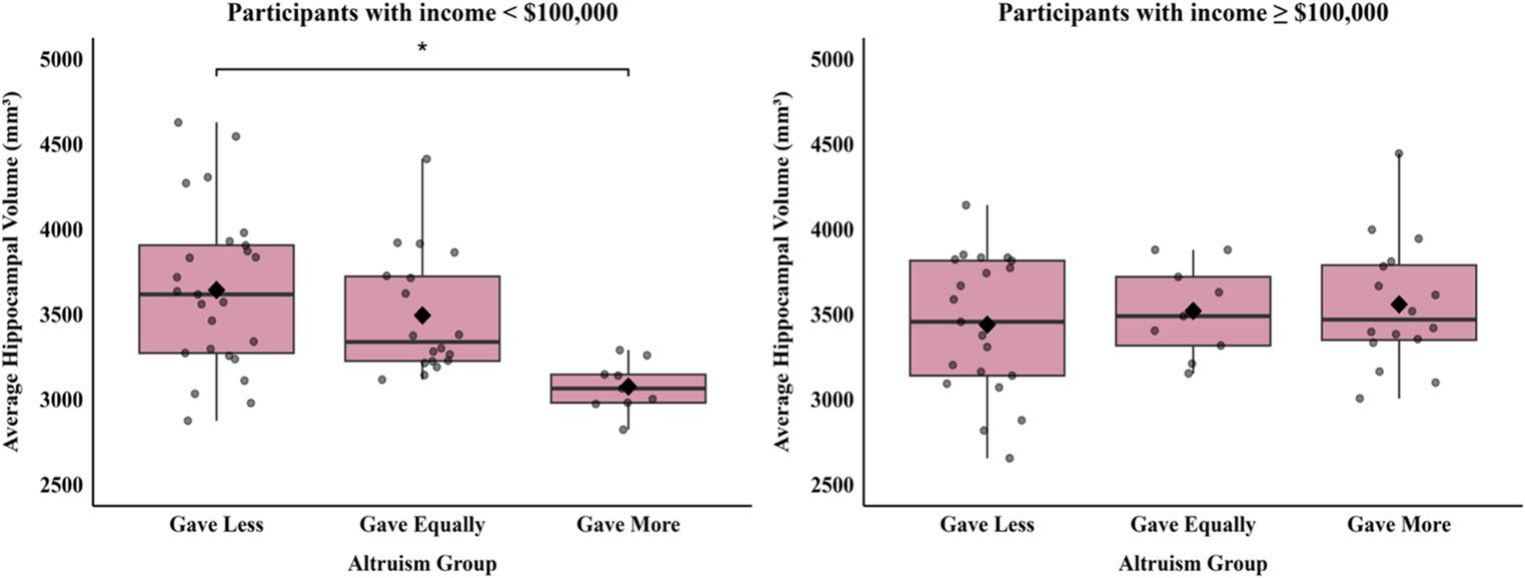
In the lower income group (<$100,000), participants in the *GM* group had significantly smaller hippocampal volume (M difference = –376.80, 95% CI [–674.67, –78.93], *p* = .04) compared to those in the *GL* group. No significant difference was observed between the *GM* and *GE* groups (M difference = –311.50, 95% CI [–609.37, –13.63], p = .10) or between the *GE* and *GL* groups (M difference = –65.30, 95% CI [–288.85, 158.25], *p* = .83). No significant differences were observed in participants in the higher income group ($100,000+)

Post-hoc analyses exploring a potential interactive effect of income and altruism group on ROI metrics revealed a significant interaction on hippocampal volume (F = 4.35, *p* = 0.02) but not other ROIs. In models stratified by income group (see Table 2 for group characteristics), there was a significant difference in hippocampal volume between altruism groups only within the lower income group (less than $100,000; F = 3.31, *p* = 0.045), such that participants in the *GM* group had significantly smaller hippocampal volume (M difference = − 376.80, 95% CI [− 674.67, − 78.93], *p* = 0.04) than those in the *GL* group (Fig. 1). To determine whether general cognitive functioning accounted for the observed association, we reran the model including performance on the Mini-Mental State Exam as a covariate. The difference between the *GM* and *GL* group remained significant, suggesting that the association was not driven by general cognition. Given the significant interaction between income and hippocampal volume, exploratory analyses stratified by income group were conducted. No significant group differences (surviving FDR correction) were observed in the lower income group. In the higher income group, there was a significant difference in thickness in the banks superior temporal sulcus (banks STS) (*p* = 0.001, FDR corrected = 0.03) between altruism groups. Participants in the *GM* group had significantly greater thickness of the banks STS (M difference = − 0.137, 95% CI [–0.205, − 0.069], *p* = 0.001) compared to those in the *GE* group. Participants in the *GL* group had marginally greater thickness of the banks STS (M difference = − 0.081, 95% CI [–0.148, − 0.014], *p* = 0.050) compared to those in the *GE* group (Supplementary Fig. 2).

**Table 2:** Higher versus lower income group characteristics

Variable	Higher Income (*N* = 46)	Lower Income (*N* = 53)	Higher vs. Lower (*p*-value)
Age, M (SD)	67.22 (6.96)	69.75 (7.17)	*p* = 0.08
Sex (% female)	80%	75%	*p* = 0.73
Education, M (SD)	17.48 (1.68)	15.92 (2.18)	***p*** **< 0.01**
Race			*p* = 0.24
% White	76%	74%	
% Asian	17%	13%	
% Black	2%	8%	
% Other	5%	0%	
% Unknown	0%	5%	
Ethnicity			*p* = 0.37
% Hispanic	2%	8%	
% Non-Hispanic	98%	92%	
MMSE, M (SD)	28.54 (1.52)	28.45 (1.47)	*p* = 0.76
TPJ thickness, M (SD) (mm)	2.36 (0.13)	2.29 (0.16)	***p*** **= 0.02**
vmPFC thickness, M (SD) (mm)	2.20 (0.10)	2.18 (0.10)	*p* = 0.31
dlPFC thickness, M (SD) (mm)	2.16 (0.13)	2.11 (0.17)	*p* = 0.06
Hippocampal volume, M (SD) (mm)^3^	3497.21 (370.76)	3492.68 (436.85)	*p* = 0.95
Entorhinal cortical thickness, M (SD) (mm)	2.61 (0.25)	2.57 (0.23)	*p* = 0.44

Differences between groups were assessed using One-Way ANOVA for continuous variables and Chi-square or Fisher's Exact Test for categorical variables. *Models assessing group differences in hippocampal volume were adjusted for intracranial volume

*MMSE* Mini-Mental State Exam; Higher Income $100,000+; Lower Income <$100,000; *TPJ* Temporoparietal junction; *vmPFC* ventromedial prefrontal cortex; *dlPF*C dorsolateral prefrontal cortex

Post-hoc exploratory analyses investigating potential differences between altruism groups in all cortical thickness and subcortical ROIs revealed no significant differences in the whole sample after FDR correction.

## Discussion

Findings from the current study provide novel information about associations between financial altruism and brain structure in older adults free from dementia. While no significant associations between financial altruism and structural ROIs were observed in the entire sample, in older adults with a reported income of <$100,000, those who exhibited greater financial altruism (i.e., those who gave more to an anonymous individual than they kept for themselves) had significantly lower volume of the hippocampus compared to those who kept more for themselves. This suggests that financial altruism in the context of lower income, but not financial altruism in general, may reflect suboptimal brain aging, and potentially greater risk of conversion to AD. If lower hippocampal volume is associated with worse financial judgment, as suggested by the present findings, this may help explain previously reported associations between increased financial exploitation vulnerability with AD risk (Boyle et al., 2019; Kapasi et al., 2021).

In the context of prior findings, our results suggest relationships between brain structure and financial altruism may differ between younger and older adults. We did not observe any associations between altruism and either of the previously implicated ROIs from the young adult literature (temporoparietal junction, dlPFC) (Morishima et al., 2012; Yamagishi et al., 2016). Rather, volume of the hippocampus appears most relevant to financial altruism in older adults. However, as noted above, this relationship was only present in those with a reported income of <$100,000. The idea that financial altruism itself is not indicative of suboptimal brain aging is further supported by our finding that, in the higher income group, participants who gave more money to the anonymous individual had *greater* thickness in the banks STS compared to older adults who split the money equally.

The mechanisms through which hippocampal volume and financial altruism are associated warrant further study. One possibility is that age-related neuropathological changes in this brain region may make it difficult to recall past experiences that would inhibit altruistic tendencies (e.g., experiences of unreciprocated cooperation) or to envision negative consequences of giving money away (e.g., greater financial strain). In support of these theories, hippocampal activity has been associated with experiences of unreciprocated cooperation (Rilling et al., 2008) and episodic future thinking (Schacter et al., 2017).

Psychological factors related to financial context may help explain why this association was specifically observed in older adults with lower reported income. Prior research suggests that financial scarcity and instability can shift decision making toward greater reliance on immediate situational or affective factors (e.g., empathic concern or social desirability), rather than on deliberative, future-oriented processes (Sheehy-Skeffington, 2020). Because the hippocampus plays a key role in integrating past experiences and future planning, age-related vulnerability in this region may have a greater impact on decision making in older adults with lower income for whom immediate considerations may be more salient.

There are several limitations to the present study. First, participants were predominantly White, female and highly educated which limits generalizability. Second, although our measure of income captured a broad range of active and passive income, it may have missed important aspects of socioeconomic status (e.g., savings). Third, while it was the optimal approach based on the observed distributions, treating altruism as a categorical variable reduced statistical power. Given that altruism was characterized using a single response measure, it is also unclear how generalizable this measure is to real-world behavior or how reliable it is. The development of ecologically valid and reliable measures of financial altruism is an important direction for future research. Fourth, due to the cross-sectional nature of the data, we cannot draw any conclusions about causality. While we hypothesize that the observed relationships between brain macrostructure and financial altruism are a result of regional atrophy, it is possible that the relationships exist prior to any neurodegeneration or that they are explained by a third variable. Longitudinal studies with repeated decision making measures and brain imaging will be necessary to infer causality. Finally, our study lacks AD biomarkers (e.g., β-amyloid and tau). In vivo markers of AD neuropathology will be critically important to support our notion that lower hippocampal volume is related to AD neuropathology. Our group is currently in the process of addressing these limitations through the collection of longitudinal data and plasma-based AD biomarkers in a subset of these participants. Investigating associations between altruism and functional connectivity is another important future direction, as connectivity changes, particularly within the Salience and Default Mode Network, have been observed early in preclinical AD and are associated with cognitive processes relevant to financial altruism (e.g., emotional empathy, perspective taking, future forecasting) (Buckner et al., 2008; Chow et al., 2022; Sheline & Raichle, 2013). Notable strengths of the study include the use of high-resolution 7 Tesla data which may contribute to more precise brain region measurements and the novel investigation of associations between brain macrostructure and financial altruism measured via a behavioral economics measure.

## Conclusions

Findings from the current study suggest greater financial altruism in the context of lower income may be associated with lower hippocampal volume. This may help explain previously observed associations between higher financial exploitation vulnerability and increased risk of conversion to AD. Longitudinal studies with AD biomarker data available are needed to further support this notion.

## Supplementary Information

Supplementary Material 1.

## Data Availability

The dataset generated and analyzed during the current study is available from the corresponding author on reasonable request.
