# Supplementary material for: Brain macrostructure correlates of financial altruism in older adults without dementia
Source: Brain Imaging Behav. 2026 Mar 17;20(2):51. doi: 10.1007/s11682-026-01133-x (PMC12996392; doi:10.1007/s11682-026-01133-x)
Supplement: Supplementary file 1 — Supplementary Material 1. [file 11682_2026_1133_MOESM1_ESM.docx]

**Supplementary Materials**

***Supplementary Figure 1: Differences in vmPFC thickness by altruism group, stratified by age***

***
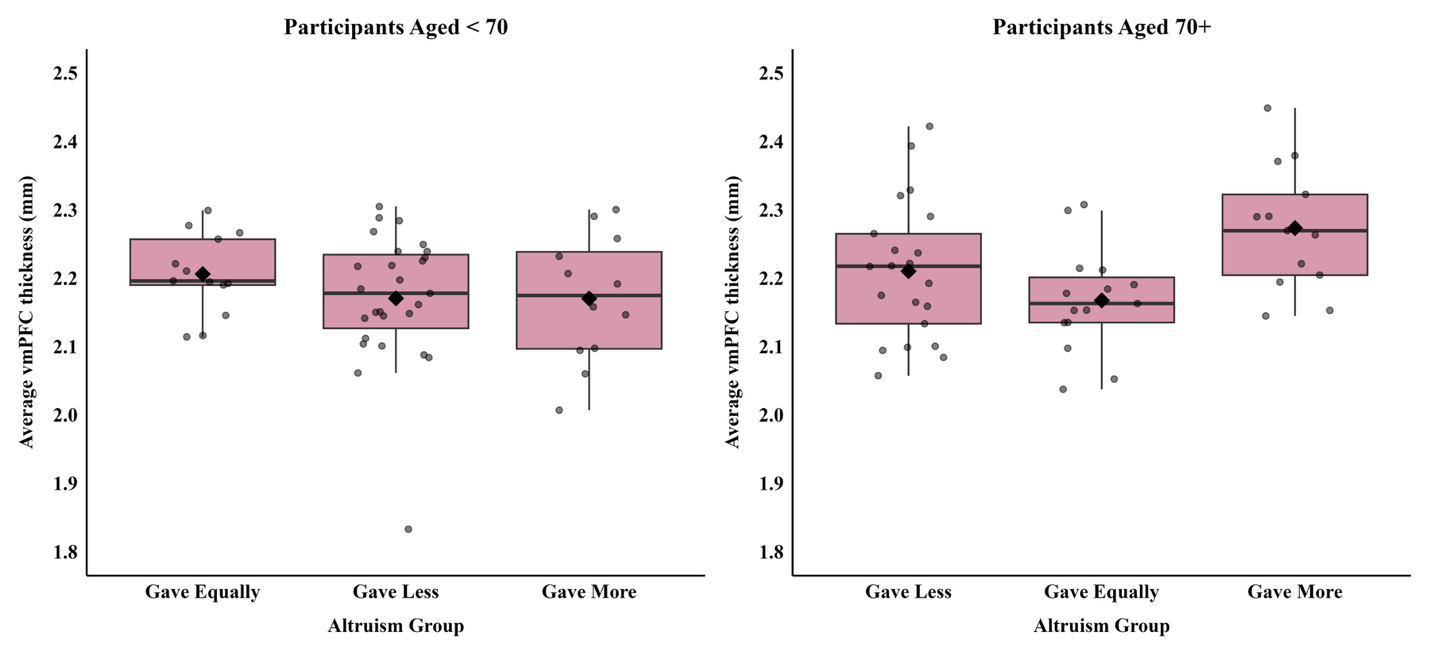
***

***Supplementary Figure 1*** vmPFC = ventromedial prefrontal cortex. In participants aged 70 and above, those in the *Gave More* group had marginally greater thickness of the vmPFC (M difference = 0.092, 95% CI [0.015, 0.169], *p* = .054) compared to those in the *Gave Equally* group. No other trend level or significant differences were observed in either group.

***Supplementary Figure 2: Differences in banks STS thickness by altruism group, stratified by income***


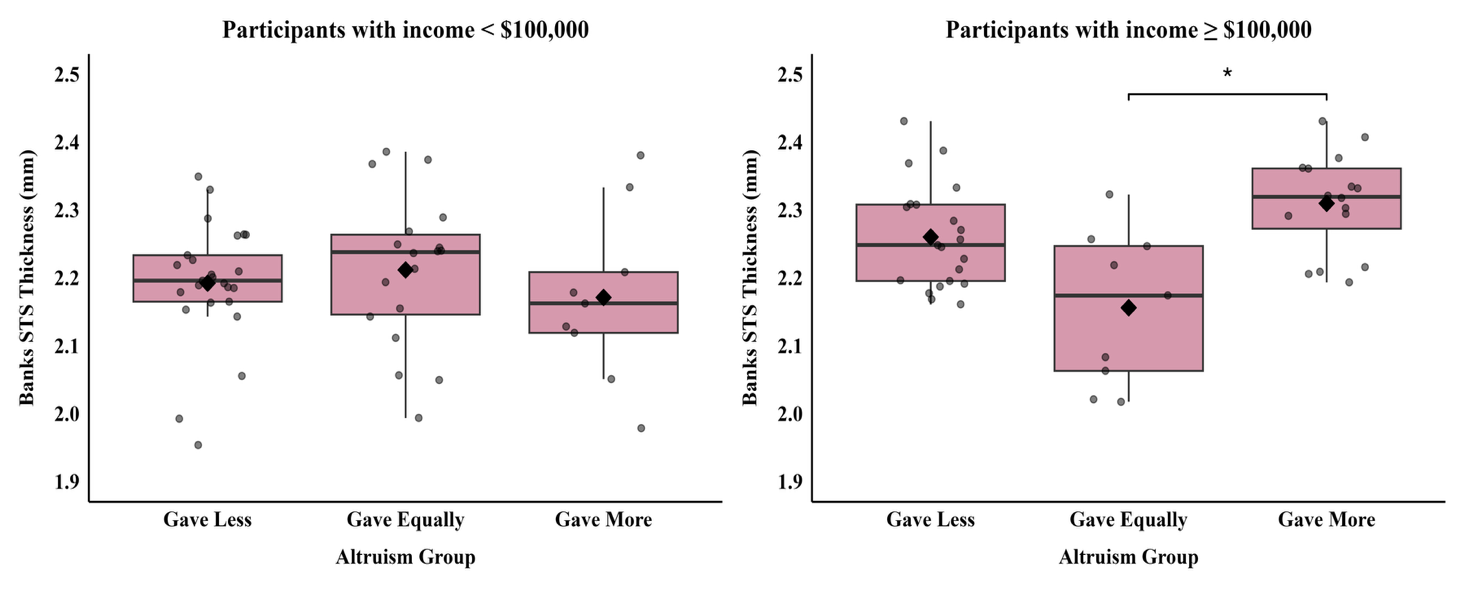


***Supplementary Figure 2*** Banks STS *=* banks superior temporal sulcus. In the higher income group ($100,000 +), participants in the *GM* group had significantly greater thickness of the banks STS (M difference = –0.137, 95% CI [–0.205, –0.069], *p* = .001) compared to those in the *GE* group. Participants in the *GL* group had marginally greater thickness of the banks STS (M difference = –0.081, 95% CI [–0.148, –0.014], *p* = .050) compared to those in the *GE* group. No significant differences were observed between the *GM* and *GL* groups (M difference = –0.056, 95% CI [–0.109, –0.004], *p* = .089). No significant differences were observed in participants in the lower income group (< $100,000)
